# Cell Penetrable Humanized-VH/V_H_H That Inhibit RNA Dependent RNA Polymerase (NS5B) of HCV

**DOI:** 10.1371/journal.pone.0049254

**Published:** 2012-11-08

**Authors:** Kanyarat Thueng-in, Jeeraphong Thanongsaksrikul, Potjanee Srimanote, Kunan Bangphoomi, Ornnuthchar Poungpair, Santi Maneewatch, Kiattawee Choowongkomon, Wanpen Chaicumpa

**Affiliations:** 1 Department of Microbiology and Immunology, Faculty of Veterinary Medicine, Faculty of Science, Kasetsart University, Bangkok, Thailand; 2 Graduate Program, Faculty of Allied Health Sciences, Thammasat University, Pathumthani, Thailand; 3 Department of Biochemistry, Faculty of Science, Kasetsart University, Bangkok, Thailand; 4 Department of Research and Development, Faculty of Medicine Siriraj Hospital, Bangkok, Thailand; 5 Department of Molecular Tropical Medicine and Genetics, Faculty of Tropical Medicine, Mahidol University, Bangkok, Thailand; 6 Department of Parasitology, Faculty of Medicine Siriraj Hospital, Bangkok, Thailand; The University of North Carolina at Chapel Hill, United States of America

## Abstract

NS5B is pivotal RNA dependent RNA polymerase (RdRp) of HCV and NS5B function interfering halts the virus infective cycle. This work aimed to produce cell penetrable humanized single domain antibodies (SdAb; VH/V_H_H) that interfere with the RdRp activity. Recombinant NS5BΔ55 of genotype 3a HCV with *de novo* RNA synthetic activity was produced and used in phage biopanning for selecting phage clones that displayed NS5BΔ55 bound VH/V_H_H from a humanized-camel VH/V_H_H display library. VH/V_H_H from *E. coli* transfected with four selected phage clones inhibited RdRp activity when tested by ELISA inhibition using 3′di-cytidylate 25 nucleotide directed *in vitro* RNA synthesis. Deduced amino acid sequences of two clones showed V_H_H hallmark and were designated V_H_H6 and V_H_H24; other clones were conventional VH, designated VH9 and VH13. All VH/V_H_H were linked molecularly to a cell penetrating peptide, penetratin. The cell penetrable VH9, VH13, V_H_H6 and V_H_H24 added to culture of Huh7 cells transfected with JHF-1 RNA of genotype 2a HCV reduced the amounts of RNA intracellularly and in culture medium implying that they inhibited the virus replication. VH/V_H_H mimotopes matched with residues scattered on the polymerase fingers, palm and thumb which were likely juxtaposed to form conformational epitopes. Molecular docking revealed that the antibodies covered the RdRp catalytic groove. The transbodies await further studies for *in vivo* role in inhibiting HCV replication.

## Introduction

The NS5B protein has RNA-dependent RNA polymerase (RdRp) activity which is pivotal for *de novo* RNA synthesis of hepatitis C virus (HCV). The protein is an attractive target of developing anti-HCV agents [1]. Similar to other polymerases, the NS5B resembles human right hand structure consisting of finger, thumb, and palm domains [1]. The polymerase active site is located in the palm [1]. NS5B acquires two different crystal forms: active closed-form-I and inactive open-form-II [1]. The closed conformation mediated by anchoring of ?1 and ?2 subdomain loops of fingers to the thumb is believed to regulate entering of RNA template and ribonucleotide (rNTP) substrate into the catalytic cavity during RNA replication [2]. NS5B lacking a hydrophobic C-terminal 55 amino acid residues (NS5BΔ55) has higher polymerase activity than the full-length NS5B [3].

There is no vaccine against HCV infection. Combined pegylated-interferon (PEG-IFN) and ribavirin is used for intervening of the chronic hepatitis C progression to the end stage liver diseases including liver cirrhosis and hepatocellular carcinoma [4]. Rationales are to enhance the host immunity and inhibit the viral RNA synthesis. Weekly IFN injection and daily oral ribavirin are necessary throughout the 24–48 week treatment course in order to expect effectiveness [4]. Even with such intensive treatment, the success rate is only about 50% due to tolerance of some HCV genotypes (1 and 4) [5]. Many patients do not comply with this regimen, partly because of the severe adverse side effects. Moreover, the treatment cost is beyond affordability of many infected individuals of the developing part of the world where HCV infection is a real problem. As such, novel anti-HCV agent with improved treatment efficacy and safety and less expensive warrants development. Recently, telaprevir and bocepprevir which are HCV protease inhibitors have been approved by US FDA [6] but these drugs are not yet widely available.

Recently, sera of camelids were found to contain not only the conventional four chain-immunoglobulin G (IgG) but also heavy chain antibody (HCAb) which each molecule consists of heavy (H) chain homodimers. The HCAb is soluble in serum in spite of the fact that the H chains do not have the linked light (L) chain partners. This is because the HCAb has mutated some hydrophobic amino acids at the former interface between the variable heavy chain domain (VH) and the variable light chain domain (VL) to be more hydrophilic; thus reducing aggregation [7]–[9]. This area is located on immunoglobulin framework-2 (FR2) of the antigen binding domain of HCAb, designated V_H_H in order to differentiate from the VH of the conventional four chain antibody. The V_H_H FR2 area contains a tetrad amino acid hallmark, *i.e*., F/Y42, E49, R/C50 and G/L52 which substitute for V42, G49, L50, and W52 of the conventional VH [7]–[9]. Besides, the third complementarity determining region (CDR3) of the V_H_H is exceptionally long and can extend to cover the FR2 hydrophobic region; thus increasing solubility of the V_H_H [7]–[9]. Recently humanized-V_H_H phage display library was constructed from naïve camel immunoglobulin genes. The camel VH/V_H_H coding genes were humanized by using human primer directed PCR amplification [10]. Phage clones secreted V_H_H specific to botulinum neurotoxin were selected from the library. One V_H_H (V_H_H17) neutralizes the zinc metalloproteinase activity of botulinum neurotoxin type A by inserting its CDR3 into the emzymatic groove of the target enzyme and blocked specifically the catalytic enzymatic activity [10]. This functional mechanism of antibody has never been possible by large molecular sized conventional antibody. More recently, humanized-camel VH/V_H_H that bound specifically to Cobra (*Naja kaouthia*) venom phospholipase A2 and inhibited the enzyme activity was reported [11]. It is now generally accepted that the V_H_H is a potent enzyme inhibitor [12]. In this study, cell penetrable humanized VH/V_H_H, synonym single domain antibodies (SdAb), that bound specifically to HCV NS5B and interfered with the native RdRp catalytic activity inside the HCV infected cells leading to inhibition of the HCV replication were produced. To our knowledge this is the first report on HCV polymerase neutralization by cell penetrable humanized-VH/V_H_H.

## Materials and Methods

### Recombinant NS5BΔ</emph>55 Protein

Complementary DNA (cDNA) was synthesized from RNA extracted from the serum of patient infected with genotype 3a HCV (kindly provided by Professor Yong Poovorawan, Chulalongkorn Hospital, Bangkok) which is the predominant serotype. The cDNA was used as a template for amplification of *NS5BΔ55* cDNA by polymerase chain reaction (PCR). Oligonucleotide primers specific to nucleotide sequence coding for HCV NS5BΔ55 protein were designed from the genotype 3a HCV nucleotide sequence of the database (GenBank NC_009824). The PCR amplicon was cloned into pET23b^+^ vector between *EcoR*I and *Xho*I sites and the recombinant plasmid was introduced into BL21 (DE3) *E. coli*. Transformed *E. coli* was grown and induced to over-express the recombinant protein by 0.2 mM isopropyl-β-D-1-thiogalactopyranoside (IPTG). The recombinant NS5BΔ55 was purified from the bacterial lysate by using Ni-NTA beads (Invitrogen) and verified by gel-based liquid chromatography-tandem mass spectrometry [13].

### RdRp Activity of the Recombinant NS5B*Δ*55

Enzyme linked immunosorbent assay (ELISA) was used for determining RdRp activity of the NS5B*Δ*55 by detecting an incorporation of biotinylated-cytosine triphosphate (CTP) (Invitrogen) into an RNA template in the presence of the NS5B*Δ*55. The SLD3 RNA (5′ GGGCUUGCAUAGCAAGUCUGAGACC 3′) [14] was used as RNA template and the procedure described previously [15] was followed with modification. The 5′ end of the SLD3 RNA (800 pM) was attached covalently to the surface of the Nucleolink module (Thermo Scientific Nunc, UK) *via* carbodiimide condensation [16]. Polymerase reaction mixture (80 *µ*L) (300 nM of NS5B*Δ*55; 20 mM sodium glutamate, pH 8.2; 4 mM MgCl_2_; 12.5 mM DTT; 0.5% (v/v) Triton X-100; 2 mM MnCl_2_; 40 units RNase inhibitor; 200 *µ*M each ATP, UTP, GTP, and biotinylated-CTP) was added to the SLD3 RNA coated well and incubated at 37°C for 2 hours. Polymerase reaction mixture containing heparin (2 *µ*M) which is polymerase quencher [17] was included in the assay as the RdRp inhibition control. Non-incorporated rNTPs were washed away before adding with streptavidin-horseradish peroxidase (HRP) conjugate (Southern Biotech, USA), followed by 2,2′-azino-di (3-ethylbenzthiazoline-6-sulfonate (ABTS) substrate (KPL, USA). Wells containing buffer and normal BL21 (DE3) *E. coli* lysate were included as blank and negative control, respectively. Optical density at absorbance 405 nm (OD_405 nm_) of the content of each well was determined.

### Humanized-camel VH/V_H_H Phage Display Library

The library was constructed previously [10] using total RNA extracted from peripheral blood mononuclear cells of a naive male camel (*Camelus dromedarius*) and messenger RNA (mRNA) was reverse transcribed to cDNA. The gene fragments encoding variable domains of the camel VH/V_H_H were PCR amplified using the cDNA as template and 14 forward and 3 reverse degenerate primers designed from all families of human immunoglobulin genes [18]. The human primer directed-camel *vh/v_h_h* cDNA amplicons were ligated into a pCANTAB5E phagemid vector and introduced into competent TG1 *E. coli* cells. The complete phage particles displaying humanized-camel VH/V_H_H with integrated *vh/v_h_h* in the phage genomes were rescued from by co-infecting the *vh/v_h_h*-phagemid transformed *E. coli* with a helper phage.

### Phage Biopanning and Preparation of the Humanized-VH/V_H_H

A single round phage biopanning for selecting phage clones that displayed NS5B*Δ*55 bound-VH/V_H_H was performed as described previously [10] using 10* µ*g of purified NS5BΔ55 as the panning antigen. Antigen bound phages were supplemented with log phase grown HB2151 *E. coli*. The phagemid transformed *E. coli* clones were selected on LB agar plate containing 100 *µ*g/mL ampicillin and 2% glucose. *E. coli* clones carrying recombinant *vh/v_h_h*-phagemids were screened by PCR using phagemid specific *R1* and *R2* primers [18]. Selected clones were grown individually under 0.5 mM IPTG induction and VH/V_H_H proteins in bacterial lysates were partially purified by ion exchange (DEAE) column chromatography. Amount of VH/V_H_H in each preparation was standardized.

### Specific Binding of VH/V_H_H to NS5B*Δ*55

Specific binding of VH/V_H_H to NS5B*Δ*55 was determined using indirect ELISA and Western blot analysis (WB) [10], [18]. For ELISA, one *µ*g of NS5B*Δ*55 and antigen control, i.e., bovine serum albumin (BSA) was immobilized separately in wells of an ELISA plate. After blocking the empty sites on well surface with 3% BSA in PBS, standardized VH/V_H_H contained in lysates of transformed *E. coli* were added to appropriate wells and kept at 37°C for 1 hour. Unbound VH/V_H_H were removed; the bound VH/V_H_H in each well was detected by adding rabbit anti-E tag antibody (Abcam, UK), goat anti-rabbit immunoglobulin-HRP conjugate (Southern Biotech), and ABTS substrate (KPL), respectively, with washing with phosphate buffered saline, pH 7.4 containing 0.5% Tween-20 (PBST) between steps. *E. coli* clones which their lysates gave OD_405 nm_ to NS5BΔ55 two times higher than to the BSA were selected.

For WB, NC strip blotted with SDS-PAGE separated-NS5B*Δ*55 was blocked with 5% skim milk in Tris buffered saline (TBS) and kept at 25°C for 1 hour. After washing with TBS containing 0.05% Tween-20 (TBST), the NC strip was incubated with *E. coli* lysate containing standardized VH/V_H_H at 25°C for 1 hour. The NS5BΔ55-VH/V_H_H reactive band was revealed by using rabbit anti-E tag antibody (Abcam), goat anti-rabbit immunoglobulin-alkaline phosphatase (AP) conjugate (Southern Biotech) and BCIP/NBT substrate (KPL).

### ELISA Inhibition for Screening VH/V_H_H that Inhibited NS5BΔ55 RdRp Activity

Test mixtures, i.e., partially purified VH/V_H_H mixed individually with NS5B*Δ*55, and control mixtures, i.e., NS5B*Δ*55 mixed with irrelevant V_H_H that specific to botulinum neurotoxin type A, V_H_H17 [10] (background inhibition control) and NS5B*Δ*55 mixed with antibody diluent (negative inhibition control or blank) were prepared. The mixtures were added separately to ELISA wells containing immobilized SLD3 RNA template. The polymerase reaction mixture prepared as above was added to each well and the ELISA procedure was similarly completed. OD_405 nm_ of the content of the test and control wells were measured against blank. Less OD_405 nm_ of the tests compared to the background and negative inhibition controls indicated that the VH/V_H_H could neutralize specifically the NS5B*Δ*55 RdRp activity.

### Production of Cell-penetrable VH/V_H_H

Gene sequence coding for the VH/V_H_H that inhibited the NS5BΔ55 RdRp *in vitro* was subcloned to *PEN-pET23b^+^* plasmid backbone [19] in order to produce cell-penetrable VH/V_H_H. The *vh/v_h_h* sequences were cloned into the recombinant plasmid vector at downstream of the DNA sequence coding for a 16 amino acid cell-penetrating peptide, i.e., penetratin (PEN) *via Sfi*I and *Not*I sites and introduced into BL21 (DE3) *E. coli*. PEN-VH/V_H_H fusion proteins were produced from transformed bacteria and purified by using Ni-NTA beads [19].

### Inhibition of HCV Replication in Huh7 Cells by Cell-penetrable VH/V_H_H

The plasmid pJFH-1 containing full-length cDNA of the JFH-1 HCV of genotype 2a (GenBank AB047639) was kindly provided by Dr. Takaji Wakita, Department of Microbiology, Tokyo Metropolitan Institute for Neuroscience, Tokyo, Japan and Professor Ralf Bartenschlager, Department of Molecular Virology, University of Heidelberg, Germany. To generate genomic HCV RNA, the pJFH-1 was linearized with *Xba*I endonuclease (Fermentas) and transcribed *in vitro* using Megascript T7 kit (Ambion, USA). The transcribed RNA (10 *µ*g) was put into Huh-7 cells (4.0×10^6^ cells) by electroporation (single pulse at 0.27 kV, 100 milliseconds) using eukaryotic electroporation mode by Electroporator® (Eppendorf). The JFH1 RNA transfected Huh7 cells were immediately transferred to 40 mL of complete (serum supplemented) DMEM and seeded into wells of 12-well culture plate (Corning), 2.0×10^5^ cells/well. After 24 hours, the cell monolayer was washed with PBS and added with complete DMEM containing 10 and 20 *µ*g of purified PEN-VH/V_H_H of individual *E. coli* clones and irrelevant PEN-V_H_H17 (specific to botulinum neurotoxin type A) [10]. Cells added with 50 nM Ribavirin +100 units of PEG-IFN served as positive inhibition control. Infected Huh7 cell monolayer added with the medium alone served as negative inhibition control.

After 5 days, total RNA was extracted from the spent culture medium and the cell monolayer using Trizol™ reagent (Invitrogen). Copy number of HCV 5′ UTR (230 bp) in 900 ng of total RNA in each extract was determined by quantitative real-time PCR [20] using 1-step Brilliant II SYBR green qRT-PCR master mix kit (Agilent Technologies, USA). A standard curve was constructed from Ct of ten-fold dilutions of the pJFH-1 carrying full-length cDNA of the JFH-1 HCV genotype 2a (ranged from 2.79×10^7^ to 0.02 copies). Ct of each sample was expressed as log_10_ of RNA copies/mL calculated from the standard curve.

Similar experiments were performed by culturing the pJFH-1 RNA transfected Huh7 cells in medium containing 20* µ*g of PEN-VH/V_H_H and PEN-V_H_H17 and ribavirin + PEG-IFN controls. The numbers of HCV foci in the pJFH-1 RNA transfected cells were enumerated by staining the cells (after washing) with mouse anti-HCV core antibody (Abcam); rabbit anti-mouse immunoglobulin-alkaline phosphatase conjugate and BCIP/NBT substrate were used as foci revelation reagents. Means ± SD of the numbers of HCV foci from 100 microscopic fields (magnification 10×20) in the PEN-VH/V_H_H treated cells were compared with the infected cells (negative inhibition control), infected cells treated with ribavirin + PEG-IFN (positive inhibition control) and the cells treated with irrelevant V_H_H17 (background inhibition control). The amounts of HCV core antigen in the culture supernatants of all treatments on day 5 were determined by using QuickTiter™ HCV Core Antigen ELISA kit (Cell Biolabs, Sandiego, USA).

### Cell Internalization Efficiencies of PEN-VH/V_H_H

To measure cell internalization efficiencies of the PEN-VH/V_H_H, Huh7 monolayer were incubated with 20 *µ*g of individual PEN-VH/V_H_H preparations for 1 hour. Cell culture supernatants were collected. Cells in individual wells were washed with plain DMEM, added with fixed volume of PBS, homogenized by freezing and thawing repeatedly and the cell lysates were collected. Each culture supernatant/cell lysate (75 *µ*L) was immobilized on ELISA well until dried. The amounts of the immobilized PEN-VH/V_H_H were quantified by indirect ELISA as described previously using mouse monoclonal anti-6x-histidine as the PEN-VH/V_H_H tracing antibody. OD_405 nm_ of the content of each wells were determined. Amount of VH/V_H_H in each preparation was determined from standard curve constructed from ELISA OD_405 nm_ of purified PEN-VH/V_H_H (ranged from 2.5 to 25 *µ*g). Duplicate experiments were performed. The % cell internalization of the PEN-VH/V_H_H was calculated from the original 20 *µ*g amount of antibody.

### LDH Assay

Cytotoxicity of individual PEN-VH/V_H_H on naïve Huh7 cells was determined by using CytoTox 96® non-radioactive cytotoxicity (LDH) assay (Promega, USA).

### Restriction Fragment Length Polymorphism (RFLP) of *vh/v_h_h* Sequences

RFLP of *Mva*I digested DNA sequences coding for the individual VH/V_H_H were determined by 14% polyacrylamide gel electrophoresis followed by ethidium bromide staining [13].

### Amino Acid Sequences, Immunoglobulin Frameworks (FRs) and Complementarity Determining Regions (CDRs) of the VH/V_H_H

The *vh/v_h_h* were sequenced and amino acids were deduced. All protein sequences were multiply aligned by ClustalW. Immunoglobulin frameworks (FRs) and complementarity determining regions (CDRs) of each VH/V_H_H were predicted by using the International Immunogenetics information system (IMGT) [21].

### Mimotope Searching

Ph.D.-12™ phage display peptide library (New England Biolabs, USA) was used to determine VH/V_H_H bound phage mimotopes as described previously [10]. The mimotope peptide sequences were deduced from the phage DNA sequences by DNAMAN software version 4.15. The mimotopes were classified into groups by using Phylogeny ClustalW [22]. The sequences of the same mimotope group were multiply aligned with HCV NS5B sequence (Accession no. NP_751928) by Kalign [23].

### ELISA Inhibition Assay for Validation of the Phage Mimotopes

Phage clones displaying the representative mimotopes of mimotope groups were propagated in ER2738 *E. coli* and the titers of the amplified phages were determined according to manufacturer’s instruction (New England Biolabs, USA). Phage mimotope preparations (50 *µ*L) at various amounts (10^6^, 10^7^ and 10^8^ plaque forming unit; pfu) were mixed individually with fixed amount of 50 *µ*L VH/V_H_H (5 *µ*g) and incubated at 37°C for 1 hour. The VH/V_H_H mixed with M13KO7 phage served as background binding control. NS5B*Δ*55 coated wells added with the VH/V_H_H served as 100% binding (maximum binding). After washing, rabbit anti-E tag antibody, goat anti-rabbit immunoglobulin-HRP conjugate and ABTS substrate were added respectively. OD_405 nm_ of the content of each wells were determined. The % ELISA inhibition was calculated:

% ELISA inhibition  =  [(OD_405 nm_ of maximum binding − OD_405 nm_ of test) ÷ (OD_405 nm_ maximum control)] × 100.

### Homology Modeling and Molecular Docking

Deduced amino acid sequences of NS5B and VH/V_H_H were subjected to basic local alignment search (BLAST). The sequences with maximum identities were used as templates for homology modeling. The constructed models were validated by using PROCHECK [24]. Three dimensional structure of the protein complex was predicted by protein docking technique. ZDOCK and RDOCK modules embedded on Discovery Studio program were used for docking. NS5B and VH/V_H_H were set as input receptor and input ligand, respectively. Each ZDOCK docking result was subjected to structure refinement by using RDOCK module. After RDOCK calculation, the dock pose with lowest RDOCK energy was analyzed for the binding interaction.

### Statistical Analysis

Means and standard deviations of three independent experiments were used for comparison between tests and controls. *P* values <0.05 of unpaired *t-*test was considered significant difference.

## Results

### Recombinant NS5BΔ55

NS5BΔ55 (∼60 kDa) of HCV genotype 3a was successfully produced and purified from the lysate of a selected transformed BL21 (DE3) *E. coli* carrying the recombinant *NS5BΔ55*-pET23b^+^ plasmids. Deduced amino acid sequence of the *NS5BΔ55* showed 98% identity to the sequence of HCV genotype 3 NS5B protein (Accession no. YP_001491557.1) and approximately 80% identity to the NS5B protein sequences of various other HCV genotypes including 1a, 1b, 6b, 6c and 6m (Figure S1). The protein was verified by LC-MS/MS as the HCV NS5B (data not shown).

**Figure 1:**
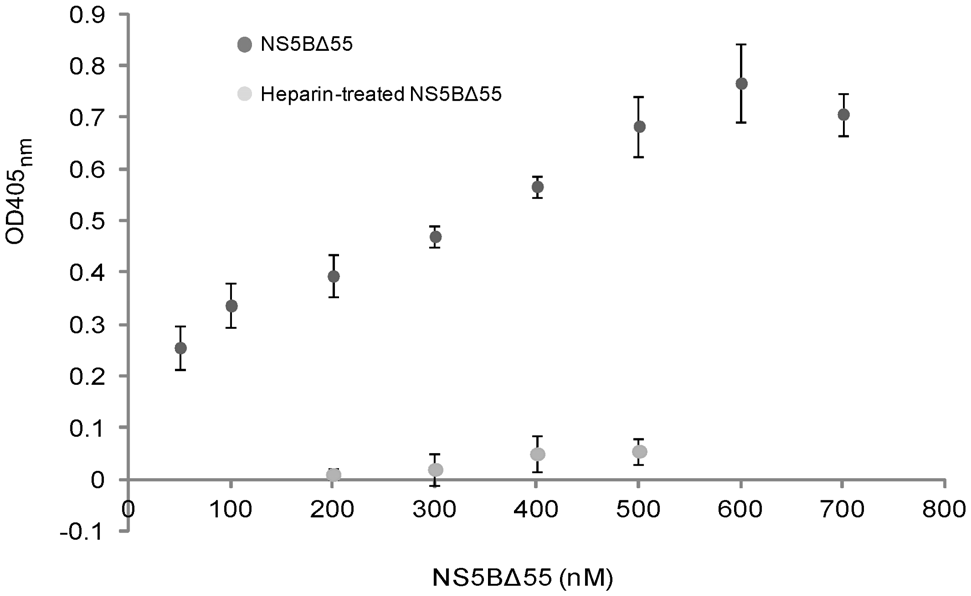
ELISA results for determining RdRp activity of recombinant NS5B*Δ*55 (300 mM) using SLD3 as an RNA template. The newly synthesized biotinylated RNA that hybridized with the immobilized SLD3 RNA templates on the ELISA well was detected using streptavidin-HRP conjugates. The OD_405 nm_ of the newly synthesized biotinylated RNA increased as the amounts of the NS3B*Δ*55 increased from 50 to 600 nm, indicating a dose-dependent RdRp activity of the recombinant protein. RdRp activity of the NS5B*Δ*55 was abolished when heparin (2 *µ*M) which is known as the polymerase trapping reagent was included into the reaction mixtures.

SLD3 RNA and biotinylated-CTP based-ELISA showed that the recombinant NS5B*Δ*55 acquired RdRp activity (Figure 1). The colorimetric values of the newly synthesized biotinylated RNA increased steadily when the amounts of NS5B*Δ*55 were increased from 50 to 600 nM. The RdRp activity of the NS5B*Δ*55 was quenched by the presence of heparin (2 *µ*M) which was known to be the polymerase trapping reagent.

### Phage Clones that Displayed NS5BΔ55-bound VH/V_H_H and the VH/V_H_H Characterization

**Figure 2:**
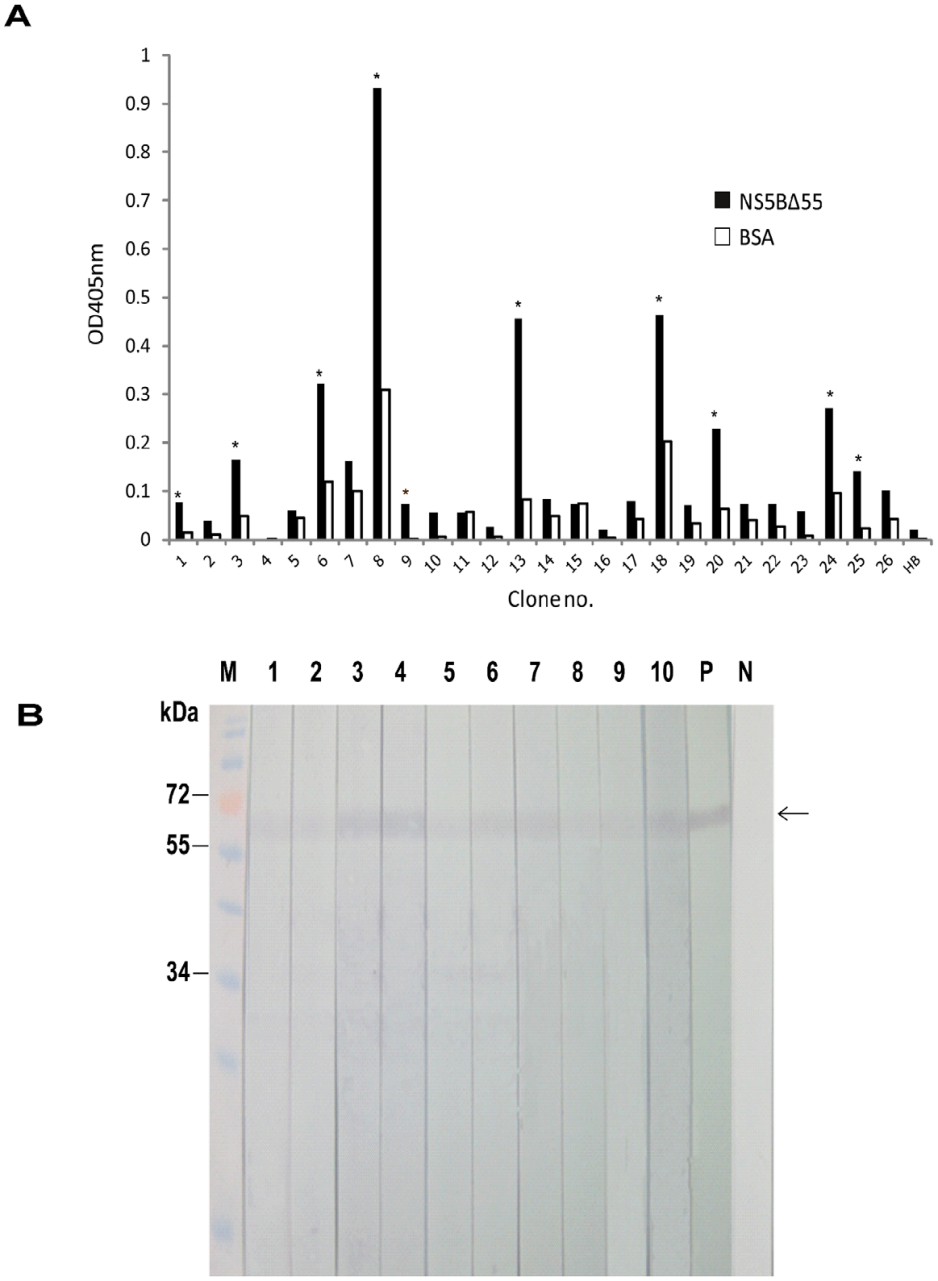
Results of experiments for selection and characterization of VH/V_H_H expressed *E. coli* clones. (A) Indirect ELISA results for detecting the binding of VH/V_H_H in lysates of 26 *vh/v_h_h*-phagemid transformed HB2151 *E. coli* clones to NS5B*Δ55. Lysates of 10 clones (no. 1, 3, 6, 8, 9, 13, 18, 20, 24, and 25) gave OD_405 nm_ to the immobilized NS5BΔ55 two times higher than to BSA control (asterisks). HB, negative VH/V_H_H control which lysate of normal HB2151 E. coli. (B) Western blot result for confirming the binding of the VH/V_H_H of the 10 ELISA positive clones to SDS-PAGE separated NS5BΔ55; VH/V_H_H of all 10 clones bound to NS5BΔ55 (arrow). Lanes 1–10, VH/V_H_H of clones no. 1, 3, 6, 8, 9, 13, 18, 20, 24, and 25, respectively. P, positive control which was SDS-PAGE separated NS5BΔ55 probed with anti-6x histidine tag. N, negative control which was SDS-PAGE separated NS5BΔ55 probed with lysate of normal HB2151 E. coli and detected by anti-E tag.*

**Figure 3:**
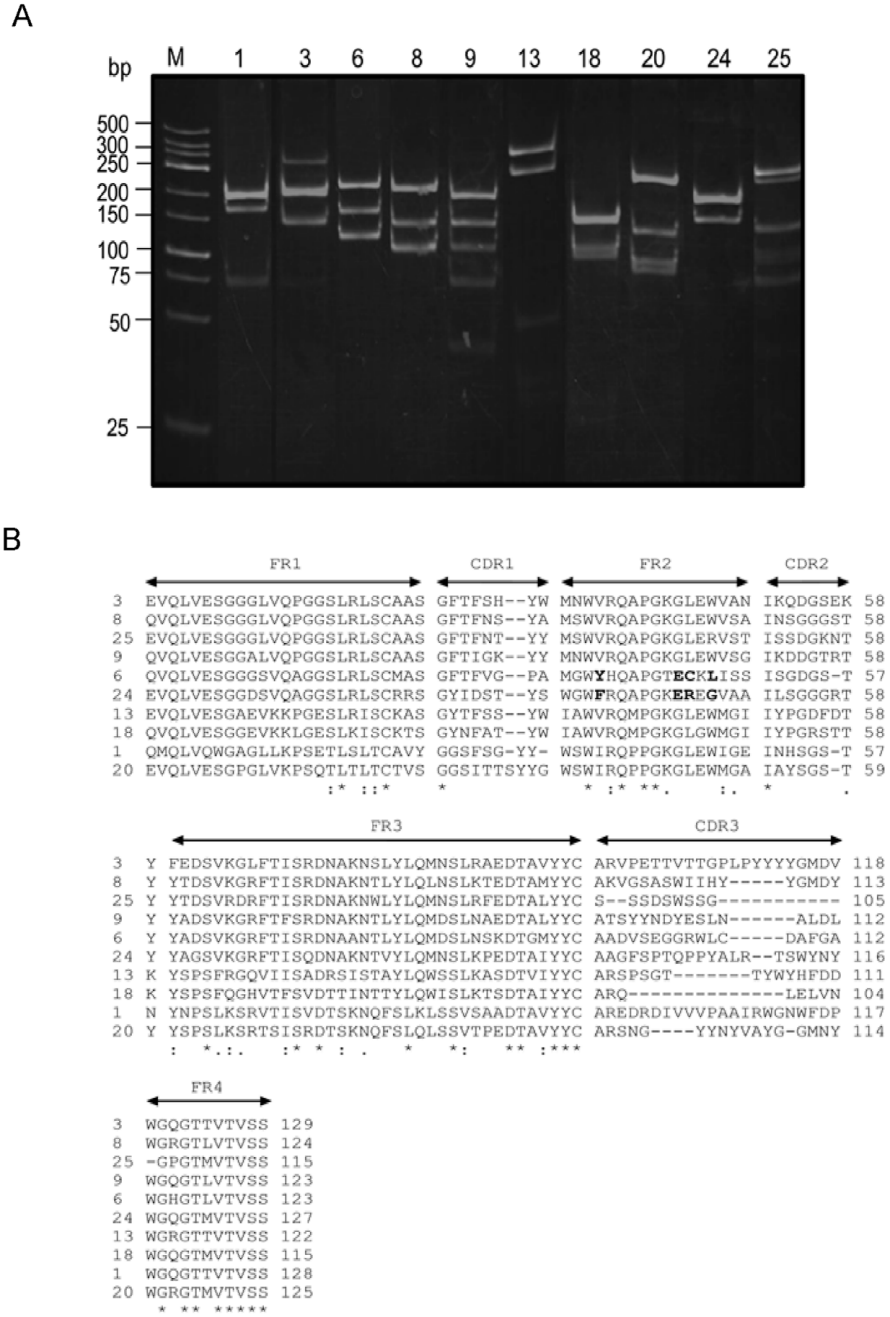
Different RFLP patterns of DNA sequences coding for the VH/V_H_H of clones no. 1, 3, 6, 8, 9, 13, 18, 20, 24, and 25, respectively. (A). Multiple alignment of amino acid sequences for determining immunoglobulin frameworks (FRs) and complementarity determining regions (CDRs) of the 10 VH/V_H_H by using the International Immunogenetics Information System sever (B). Clones no. 6 and no. 24 have the tetrad amino acid hallmark of V_H_H in FR2 (bold letters) and were designated V_H_H6 and V_H_H24; the rests were conventional VH, designated VH1, VH3, VH8, VH9, VH13, VH18, VH20, and VH25 Asterisk indicates identical amino acids; colon indicates conserved amino acid substitution; and dot indicates a semiconserved amino acid substitution.

From 40 selected HB2151 *E. coli* colonies grown on the selective agar, 29 clones were positive by PCR for *vh/v_h_h* sequences (∼600 bp) and 26 clones could express VH/V_H_H (15–25 kDa) as determined by WB. They were designated clones no. 1–26. VH/V_H_H in the lysates of 10 of the 26 clones bound to NS5BΔ55 by indirect ELISA (Figure 2A) as well as by WB (Figure 2B). The *vh/v_h_h* sequences of the 10 clones revealed 10 different DNA banding patterns (RFLP) (Figure 3A). Multiple alignments showed that all clones had different amino acid sequences especially at the CDR domains (Figure 3B). The deduced amino acid sequences of two clones had the characteristic amino acid tetrad of V_H_H; they were designated clones V_H_H6 and V_H_H24, while the other clones had conventional VH feature; thus, designated clones VH1, VH3, VH8, VH9, VH13, VH18, VH20, and VH25 [7], [10]. Sequences of these 10 humanized-camel VH/V_H_H showed high homology with human VH sequences (Table 1).

**Table 1:** Percent amino acid homology of the VH/V_H_H sequences with the closest human V region frameworks.

VH/V_H_Hclone number	Closest human V region	Percent amino acid homology with human FRs		
		FR1	FR2	FR3
VH1	AB019439 IGHV4-34*01	92.00	100.00	97.37
VH3	X92288 IGHV3-7*02	100.00	94.12	92.11
V_H_H6	AJ879486 IGHV3-23*04	86.21	41.18	78.95
VH8	AJ879486 IGHV3-23*04	96.00	100.00	84.21
VH9	HM855939 IGHV3-NL1*01	92.00	88.24	86.84
VH13	X56368 IGHV5-51*03	88.00	94.10	84.21
VH18	M99686 IGHV5-51*01	80.00	88.24	71.05
VH20	X92274 IGHV4-30-4*03	88.00	88.24	78.95
V_H_H24	Z27504 IGHV3-66*02	80.00	59.24	81.58
VH25	AJ879484 IGHV3-h*01	96.00	82.35	84.21

* Indicates allele polymorphism.

### Humanized-VH/V_H_H Mediated Neutralization of NS5BΔ55 RdRp Activity

**Figure 4:**
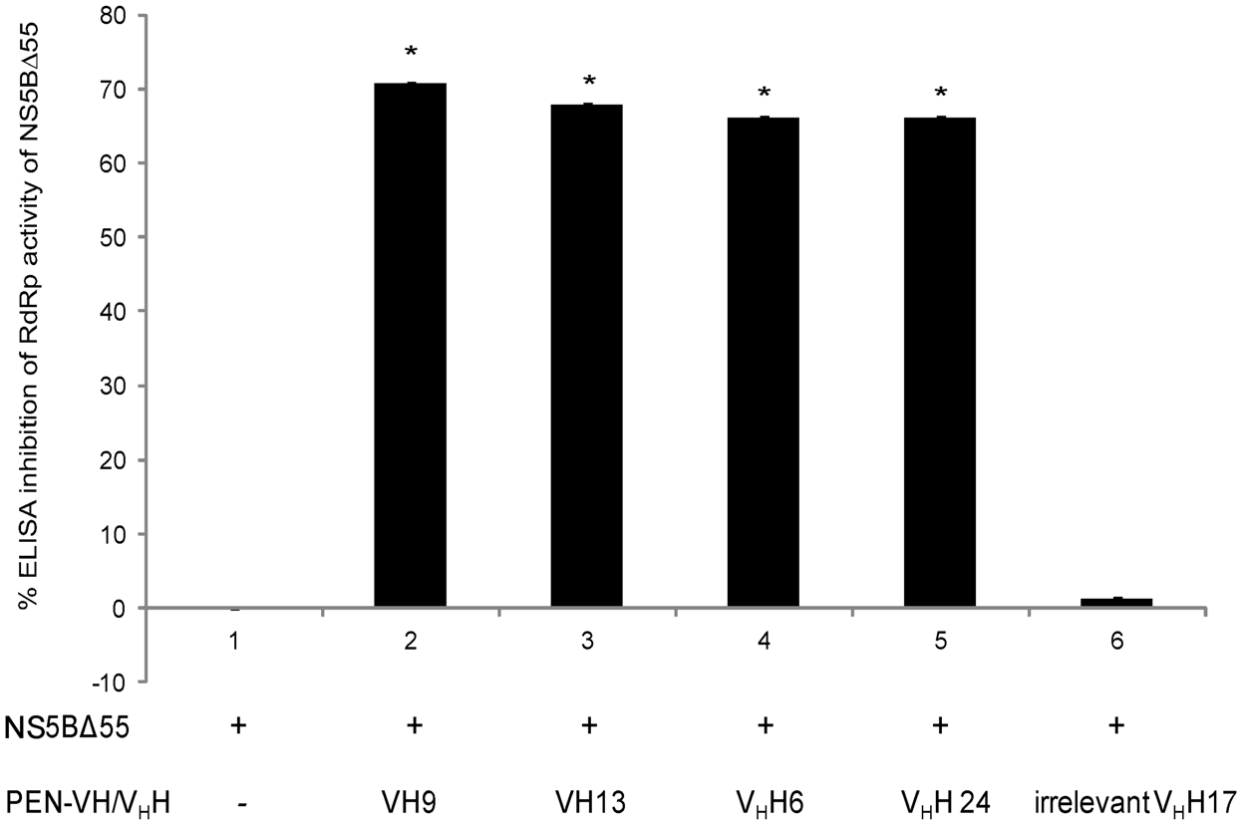
Percent ELISA inhibition of RdRp activity of NS5B*Δ55 mediated by VH9, VH13, V_H_H6, and V_H_H24 (bars 2–5, respectively).* ELISA mixture containing NS5B*Δ*55 alone (bar 1) served as negative inhibition control. Bar 6, reaction mixture containing PEN-V_H_H17 specific to botulinum neurotoxin type A [10] was included as background inhibition control. OD_405 nm_ of bars 1–6 were 0.151±0.053, 0.165±0.108, 0.175±0.076 0.174±0.078 and 0.508±0.061, respectively, while the OD of the ELISA without the SdAb was 0.514±0.111. *, different significantly from the negative inhibition control.

Partially purified VH/V_H_H of the 10 HB2151 transformed *E. coli* clones were screened for NS5BΔ55 RdRp neutralizing activity by ELISA inhibition. At 2–4 *µ*g, V_H_H6, VH9, VH13 and V_H_H24 inhibited the RdRp activity of 300 nM NS5BΔ55 by 10–69% (data not shown). The *vh/v_h_h* sequences of these clones were sub-cloned into *PEN-*pET23b^+^ backbone. The PEN-VH/V_H_H expressed from the IPTG induced transformed BL21 (DE3) *E. coli* carrying the respective plasmids were purified and tested for their ability to inhibit the NS5BΔ55 RdRp activity by the SLD3 RNA and biotinylated-CTP based-ELISA. At a molar ratio 3∶1 of antibody to NS5BΔ55, the PEN-VH9, PEN-VH13, PEN-V_H_H6, PEN-V_H_H24 and irrelevant PEN-V_H_H17 could neutralize the RdRp activity of the NS5BΔ55 (300 nM) by 70.74, 67.89, 66.01, 66.08 and 1.25%, respectively (Figure 4). The Means ± SD of the ELISA OD_405 nm_ of the wells containing the PEN-VH9, PEN-VH13, PEN-V_H_H6, PEN-V_H_H24 and irrelevant V_H_H17 were 0.151±0.053, 0.165±0.108, 0.175±0.076, 0.174±0.078 and 0.508±0.061, respectively, while the Means ± SD of the ELISA OD_405 nm_ without the SdAb was 0.514±0.111.

### Inhibition of HCV Replication in Huh7 Cells by Cell-penetrable VH/V_H_H

**Figure 5:**
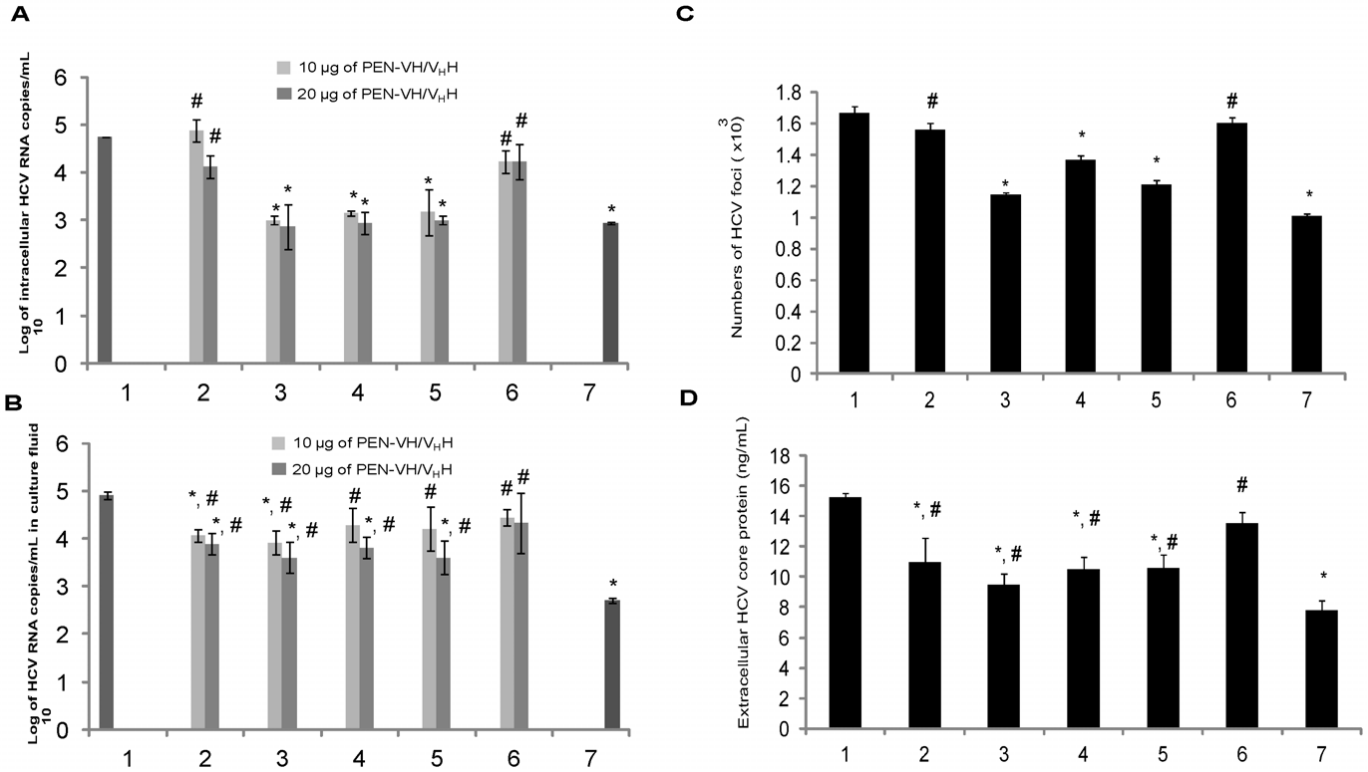
Results of qPCR for determining the log_10_ of amounts of intracellular HCV RNA (A) and released HCV RNA in culture fluids (B) of the pJFH-1 RNA transfected Huh7 cells cultured in the medium (1) which was the negative inhibition control, medium containing 10 and 20 µg of cell-penetrable PEN-VH9, PEN-VH13, PEN-V_H_H6 and PEN-V_H_H24 (2–5, respectively), medium containing 20 *µ*g of irrelevant PEN-V_H_H17 which served as the background inhibition control (6) and medium containing ribavirin + PEG-IFN which was positive inhibition control (7). Penetratin added to the cell culture medioum did not cause any inhibition of the HCV replication (data not shown). (C) Amounts of HCV core antigen (ng/mL) in cell culture supernatant of pJFH-1 transfected Huh7 cells cultured in the medium (1); medium containing 20 *µ*g of PEN-VH9, PEN-VH13, PEN-V_H_H6, and PEN-V_H_H24 (2–5, respectively); medium containing 20 *µ*g of irrelevant PEN-V_H_H17 (6); and medium containing ribavirin + PEG-IFN (7) quantified by using QuickTiter HCV core antigen ELISA kit. (D) Numbers of HCV foci in transfected Huh7 cells (1), transfected Huh7 cells exposed to 20 *µ*g of PEN-VH9, PEN-VH13, PEN-V_H_H6, PEN-V_H_H24a and irrelevant PEN-V_H_H17 (2–6, respectively) and ribavirin + PEG-IFN (7). Means ± SD of the HCV foci in 100 microscopic fields (magnification 200×) were 1.55×10^3^±41, 1.14×10^3^±18, 1.36×10^3^±29, 1.21×10^3^±29, 1.60×10^3^±35 and 1.00×10^3^±17, respectively. *, different significantly from (1); #, different significantly from (7).

The pJFH-1 RNA transfected-Huh7 cells cultured in the medium containing 10 and 20 *µ*g of VH13 had significantly less HCV RNA inside the cells (Figure 5A, bar 3) and in culture supernatants (Figure 5B, bar 3) than the transfected cells cultured in the medium alone (negative inhibition control) and the cells treated with irrelevant PEN-V_H_H17 (background inhibition control) (*p*≤0.01). Both extracellular and intracellular HCV RNA of cells exposed to 20 *µ*g of PEN-V_H_H6 and PEN-V_H_H24 were also less than both controls. Nevertheless, the amounts of the HCV RNA in the culture fluids of the cells exposed to 10 *µ*g of the two antibodies were not different from both controls. PEN-VH9 had much less inhibitory activity on the HCV RNA replication than the other three antibodies. The means ± SD of the viral RNA detected inside the cells exposed to VH13, V_H_H6 and V_H_H24 were as low as the amounts in the cells treated with ribavirin + PEG-IFN (positive inhibition control).

Numbers of HCV foci in HCV RNA transfected Huh7 cells of all treatments were expressed as means ± SD in 100 microscopic fields (magnification 200×) (Figure 5C). The numbers of the foci in the infected cells exposed to 20 *µ*g of PEN-VH13, PEN-V_H_H6, PEN-V_H_H24 and ribavirin + PEG-IFN were not different and were less than the negative and the background inhibition controls.

Figure 5D shows the amounts of HCV core antigen (ng/mL) in cell culture supernatants of pJFH-1 RNA transfected Huh7 cells. Culture fluids of the cells treated with 20 *µ*g of all tested VH/V_H_H had less antigen amounts than that of the irrelevant PEN-V_H_H17 treated cells and the cells in the medium alone.

### Cell Entering Efficiencies of the PEN-VH/V_H_H

After treating the Huh7 cells with 20 *µ*g of PEN-VH9, PEN-VH13, PEN-V_H_H6 and PEN-V_H_H24, 16.4, 17.0, 16.6 and 17.2 *µ*g of the antibodies were recovered from the cell lysates, calculated to be 82%, 85%, 83% and 86%, respectively The PEN-VH/V_H_H could not be detected in the culture supernatants.

Correlation of the intracellular amounts of the SdAbs with their inhibitory activity on the HCV replication was not clearly demonstrated (Figure 4) although the PEN-VH9 which had the least cellular entering capacity had the lowest HCV inhibitory activity.

The PEN-VH/V_H_H at the amounts up to 10 *µ*M did not cause any detectable LDH leakage from the Huh7 cells after 24 hour incubation indicating their innocuousness (Figure S3).

### VH/V_H_H Bound Phage Mimotopes and Tentative Epitopes of the VH/V_H_H on NS5B

The 12 mer peptides deduced from genomes of the phages that bound to VH/V_H_H (phage mimotopes) could be classified into several homology groups; individual mimotope peptides are shown in Table S1. Sequences of each mimotope group were aligned with NS5B sequence of the database to locate the tentative VH/V_H_H peptide epitopes on the HCV NS5B (Figure 5A). VH9 mimotopes matched with amino residues: 206–219 and 341–352 of palm and 384–395, 386–397, 413–424 and 493–504 of thumb; VH13 mimotopes matched with the residues: 20–31 of a loop interconnecting fingers and thumb, 261–272 of the α-helix that links fingers and palm, 290–301 of palm and 413–424 and 524–551 of thumb; V_H_H6 mimotopes matched with residues: 226–237 of fingers, 261–272 in α-helix that links fingers and palm, and 413–424 and 538–550 of thumb; and V_H_H24 mimotopes matched with residues: 93–104 and 95–106 of fingers and 380–401 and 491–502 of thumb. The results indicate that the VH/V_H_H bound to conformational epitopes of the NS5B.

### Inhibition of the VH/V_H_H Binding to the NS5BΔ55 by Phage Mimotopes

Representative results of the ELISA inhibition for determining the ability of phage mimotopes in inhibiting the VH/V_H_H binding to the NS5BΔ55 are shown in Figure S2. Binding of the V_H_H6 to the NS5BΔ55 was inhibited by the V_H_H6-phage mimotope groups 1 (M6-7: ALWPPNLHAWVP), 2 (M6-5: -FWSPN-HLMMNNL), 3 (M6-1:–TLHLSHWTSSAL; M6-15: HYPTTQLPHHKQ) and 4 (M6-12: GTVGRTEVSISE-; M6-16: -YSAHNYIGDSGR) implying that the mimotopes carried the amino acid residues analogous to the native HCV NS5B polymerase which validated the mimotope search results.

### Interface Binding of VH/V_H_H with the NS5B

**Figure 6:**
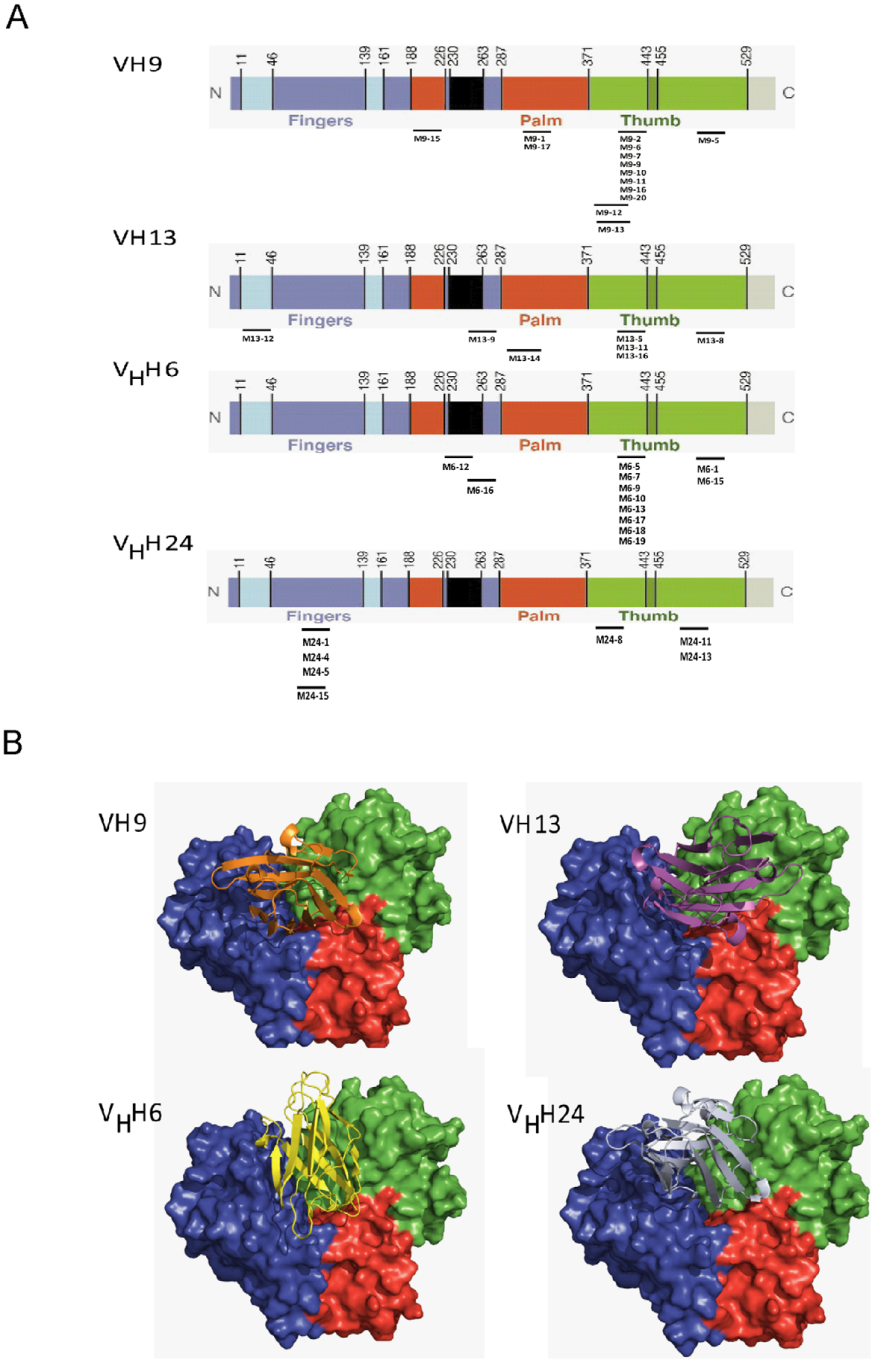
Tentative locations of amino acid residues on NS5B primary sequence matched with the respective VH9, VH13, V_H_H6 and V_H_H24 phage mimotopes, i.e., tentative epitopes of the antibodies. Fingers, palm, thumb, loop interconnecting fingers and thumb, α-helix linking palm and fingers, and β-loop insertion are colored in blue, red, green, cyan, black, and dark green, respectively. B, Hypothetical models showing binding sites of the ribbons of VH9 (orange), VH13 (magentas), V_H_H6 (yellow), and V_H_H24 (grayish blue) on molecular surface of NS5B RNA duplexes. Finger, palm and thumb of the NS5B are colored in blue, red, and green, respectively.

The amino acid sequence of NS5B genotype 3a has 73% identity with hepatitis C virus NS5B RNA polymerase (PDB code 2HAI). The Ramachandran plot of structure of NS5B was validated by using PROCHECK, and it has no residue in disallowed region (complete match). The Ramachandran plots of VH/V_H_H models are shown in Figure S4. The docked pose of NS5B and VH9, VH13, V_H_H6 and V_H_H24 are shown in Figure 6B. All antibodies interacted with all three domains of the NS5B and covered the RdRp catalytic groove.

## Discussion

There is a clinical need for safer, less expensive and more effective anti-HCV agents. The new agents should be highly tolerable to viral antigenic variation and requires shorter treatment duration than the current protocol. NS5B with inherent RdRp activity is one of the novel anti-HCV targets. Incapacitating the protein activity interrupts the HCV infectious cycle by halting replication. Many small molecular pharmacologic agents that target the NS5B RdRp have been developed and some have reached clinical trials. Nevertheless, most of them caused undesirable side effects and/or rapid selection of drug resistant HCV mutants [25]. Besides, their RdRp inhibition tends to be HCV genotype specific [26]. Alternative approach was used in this study to develop specific inhibitor of the intracellular NS5B RdRp activity.

An antibody molecule has multiple CDRs which contact many residues of either linear or conformational epitope of the target antigen. Thus, specific antibody can tolerate not only single point mutation but also multiple nucleotide mutations or even several amino acid alteration of the antigen [27]. Antibody based-immunotherapy has been practiced for treatment of viruses, intoxications, envenomation and inflammations including autoimmune disorder and allergy [28]. Intact antibody molecules or their engineered fragments with reduced immunogenicity, i.e., Fab, ScFv and SdAb have been developed in the past few decades and many have been approved for clinical use [28]. SdAb are highly potent enzyme inhibitors which directly insert CDRs, particularly CRD3, into the catalytic groove [10]; the mechanism which conventional antibody is lacking. Due to small sizes (15–20 kDa), the sdAb has higher penetrating ability and better *in vivo* tissue distribution than their larger size counterparts (150 kDa IgG or 25–35 kDa ScFv). Recently, a humanized-camel VH/V_H_H phage display library was constructed [10]. VH/V_H_H coding sequences of this library had high homology to human VH coding sequences [10]. Thus, VH/V_H_H derived from this library should have negligible immunogenicity in human recipient.

In this study, bacterially expressed recombinant NS5BΔ55 of genotype 3a HCV which retained the RdRp activity [3] was successfully produced and conveniently purified. Thus, the recombinant protein was suitable for use as an antigen in a single round phage bio-panning for selecting phage clones that bound to the protein from the established humanized-camel VH/V_H_H phage display library. The advantages of single over multiple round phage-panning had been discussed previously [10], [13]. The NS5BΔ55-bound phages recovered from the panning might be either antigen specific phages (bound to the antigen *via* the displayed VH/V_H_H) or non-specific phages (adhered to the antigen by means of phage coat proteins). Therefore, specific binding of the soluble VH/V_H_H prepared from lysates of the recombinant phagemid transformed bacteria to the NS5BΔ55 had to be verified using the indirect ELISA and WB. According to the set positive criteria of the assays, 10 *vh/v_h_h*-phagemid transformed *E. coli* clones were selected. The *vh/v_h_h* sequences of these clones had 10 different DNA banding patterns (RFLP) after digesting with *Mva*I endonuclease indicating high diversity among them. This was confirmed by DNA sequencing. Each *vh/v_h_h* was a complete coding sequence of an antigen binding variable (V) domain containing four immunoglobulin framework segments (FRs 1–4) and three CDR segments (CDRs 1–3) (Figure 3B). FR2 sequences of two clones showed characteristic amino acid tetrad of V_H_H; thus they were V_H_H. The FR2 sequences of the remaining clones had conventional VH feature. Multiple alignments revealed that all 10 VH/V_H_H sequences were diverse especially in the CDRs implying that they might bind to different epitopes of the NS5B and conferred different inhibitory efficacy on RdRp activity. When antibodies from all clones were screened at the same weight basis for their ability to inhibit the NS5B RdRp activity by the SLD3 based-ELISA inhibition, only four clones, VH9, VH13, V_H_H6 and V_H_H24 could inhibit the polymerase function while the rests were refractory. Thus these four clones were tested further for inhibition of native HCV RdRp in the hepatic cells transfected with genomic replicon of heterologous HCV, i.e., JFH1 RNA of genotype 2a, that are readily available and widely used in the HCV biology research and anti-HCV drug development [29]–[31].

Because the antibody must reach the intracellular NS5B in order to inhibit the *de novo* RdRp activity, the DNA sequences coding for all four VH/V_H_H clones were linked molecularly to a DNA sequence coding for a 16 amino acid cell penetrating peptide (penetratin, PEN) in a plasmid backbone constructed previously in our laboratory [19]. The cell penetrable VH/V_H_H (transbodies) specific to NS5B, i.e., PEN-VH9, PEN-VH13, PEN-V_H_H6 and PEN-V_H_H24, were successfully produced as bacterial inclusions by the respective transformed *E. coli*. Fortunately, after refolding all of the transbodies still retained the NS5B RdRp inhibitory activity similar to their original molecules.When added to the cell culture medium of Huh7 cells transfected with the JFH1 RNA the cell penetrable VH/V_H_H suppressed replication of the HCV RNA replicon, albeit in different degrees, as shown by reduction of both intracellular and released viral RNA copies. Quantification of the HCV phenotypes inside the respective VH/V_H_H exposed transfected cells and in the culture fluids conformed to the results on the viral RNA detection. Overall data indicate the cross-genotypic inhibitory activity of the transbodies. Unfortunately genomic replicons of other HCV genotypes are not available for testing the cross-genotype inhibition. Nevertheless, the high amino acid sequence homology was observed among NS5B proteins of different HCV genotypes deposited in the Genbank database. Moreover, all HCV genotypes share identical RdRp Dx_4–5_D and GDD motifs of the catalytic groove [32]. Thus, it is optimistically envisaged that the cell penetrable VH/V_H_H produced in this study should also cross-neutralize the RdRp activity of other heterologous HCV genotypes.

Phage peptides bound to the NS5B specific-VH/V_H_H (mimotopes) were searched for predicting the locations of the NS5B protein interacted by the antibodies which would enlighten the molecular RdRp inhibitory mechanism of the antibodies. Multiple alignments of the mimotopes indicated that the VH/V_H_H bound to discontinuous (conformational) epitopes on the NS5B molecule which the antibody contact residues scattered on the polymerase, either on thumb and palm (VH9) or on palm, thumb and finger domains) [33]. The findings by indirect ELISA inhibition that representative phage clones displaying the mimotope groups could inhibit the VH/V_H_H binding to the NS5BΔ55 indicate the amino acids on the HCV polymerase analogous to the mimotope peptides are the presumptive VH/V_H_H epitopes. The multiple contact points of the VH/V_H_H to the target NS5B would render them high tolerability to the HCV mutations [27]. It is noteworthy that the mimotopes of the clone VH9 which conferred the least HCV replication inhibition among the four antibody clones are located on the thumb and palm only, while mimotopes of the other clones interacted also with the interconnecting loop between fingers and thumb (VH13), α-helix that links fingers and palm (V_H_H6) or fingers (V_H_H24). It is known that the RdRp HCV is active under the closed configuration (forming a tunnel for template and ribonucleotides accommodation) of the polymerase protein formed by anchoring of the ?1 and ?2 loops of the fingers and the thumb [2]. Thus it is plausible that the observed higher inhibitory activity of the VH13, V_H_H6 and V_H_H24 than the VH9 on the HCV replication was due to their interference with the RdRp tunnel formation which was likely to be more readily than the VH9.

The results of homology modeling and molecular docking confirmed that the VH/V_H_H mediated interface binding to the NS5B molecule by occupying multiple areas around the RdRp template channel [34] and also covered the RdRp active site. The findings presumptively indicate that the NS5B specific VH/V_H_H suppressed the polymerase activity by preventing accessibility of the template/substrate to the enzyme catalytic cavity which consequently incapacitated the HCV RNA replication.

A unique feature that distinguishes the active HCV RdRp from the other polymerases is the closed hand conformation of the former as opposed to the openhand structure of the latter. Alteration of the closed configuration of HCV RdRp impaired the RNA synthetic activity [35]. Legacy from evolutional studies have documented the un-relatedness of eukaryotic RdRp, viral RdRp and DNA-dependent RNA polymerase (DdRp) [36]. The β’ subunit of the DdRp and eukaryotic RdRp contain a signature motif DbDGD (b is a bulk residue) which contributes to the polymerase catalytic activity *via* divalent cation coordination, whereas the core catalytic groove of HCV RdRp contains Dx_4–5_D and GDD motif in the palm domain [37]. No other similarity was detected between DdRp and RdRp [38]. Moreover, the biochemical activity of the HCV RdRp is also different enough from the host DNA polymerases. The HCV NS5B does not express in normal human cells. Thus, the HCV NS5B specific cell penetrable VH/V_H_H that interfere HCV RdRp function produced in this study should not inhibit the host cellular enzymes and should be harmless. Preliminary result of LDH assay performed on Huh7 cells indicated that the cell penetrable VH/V_H_H at the amount as high as 10 *µ*M were not toxic to the cells implying the antibody innocuousness.

## Supporting Information

Figure S1 Multiple alignments of the cloned NS5B amino acid sequence with the NS5B sequences of various HCV genotypes/subtypes of the database. The homology of the cloned sequence with the heterologous genotype/subtype sequences was approximately 80%.
(TIF)

Figure S2 Percent ELISA inhibition of the V_H_H6 binding to the NS5BΔ55 mediated by the V_H_H6-phage mimotope groups 1, 2, 3 and 4. In the assay, phages displaying V_H_H6 mimotope group 1 (M6-7: ALWPPNLHAWVP), group 2 (M6-5: -FWSPN-HLMMNNL), group 3 (M6-1:–TLHLSHWTSSAL; M6-15: HYPTTQLPHHKQ) and group 4 (M6-12: GTVGRTEVSISE-; M6-16: -YSAHNYIGDSGR) (Table S1) at 10^6^, 10^7^ and 10^8^ pfu were separately mixed with V_H_H6 (5 *µ*g) before adding to the ELSA well containing immobilized NS5BΔ55 (10 *µ*g). The % ELISA inhibition was calculated. The results indicate that the mimotopes could inhibit the V_H_H6 binding to NS5BΔ55 implying that the mimotopes carried the amino acid residues analogous to the native HCV NS5B polymerase which validated the mimotope search results.
(TIF)

Figure S3 Results of LDH assay for determining cellular toxicity of 10 *µ*M of VH9, VH13, V_H_H6 and V_H_H24 (1–4, respectively) on Huh7 cells after 24 hour incubation. Maximum LDH release control is shown in (5). All antibody preparations did not cause significant release of the LDH from the cells indicating the innocuousness of the preparations.
(TIF)

Figure S4 Ramachandran plots of the VH/V_H_H models. VH9, VH13, V_H_H6 and V_H_H24 had 2.8%, 0%, 4% and 0% of residue in disallowed region, respectively. The highest identity sequences used for protein homology modeling of VH9, VH13, V_H_H6 and V_H_H24 are sequences of PDB codes 2GCY, 2H32, 1VHP and 1F2X, respectively.
(TIF)

Table S1 Phage mimotope groups, respective 12-mer peptides displayed on the phages that bound to VH9, VH13, V_H_H6 and V_H_H24 and mimotope-matched peptides (tentative epitopes of the antibodies) on NS5B primary protein sequence.
(DOC)
