# Supplementary figures and images for: Cell Penetrable Humanized-VH/VHH That Inhibit RNA Dependent RNA Polymerase (NS5B) of HCV
Source: PLoS One. 2012 Nov 8;7(11):e49254. doi: 10.1371/journal.pone.0049254 (PMC3493538; doi:10.1371/journal.pone.0049254)

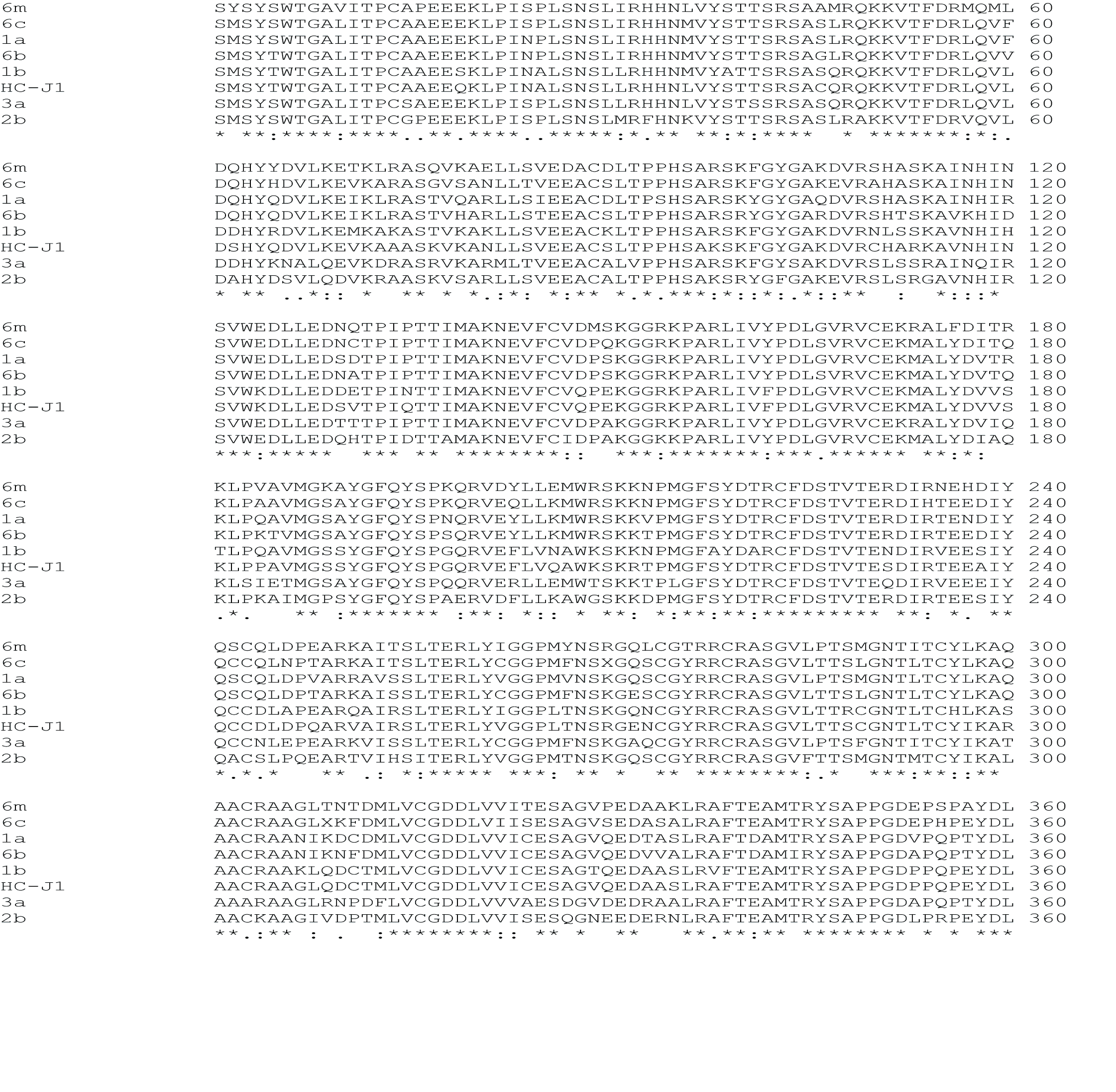

Supplement: Figure S1 — Multiple alignments of the cloned NS5B amino acid sequence with the NS5B sequences of various HCV genotypes/subtypes of the database. The homology of the cloned sequence with the heterologous genotype/subtype sequences was approximately 80%. (TIF) [file pone.0049254.s001.tif]

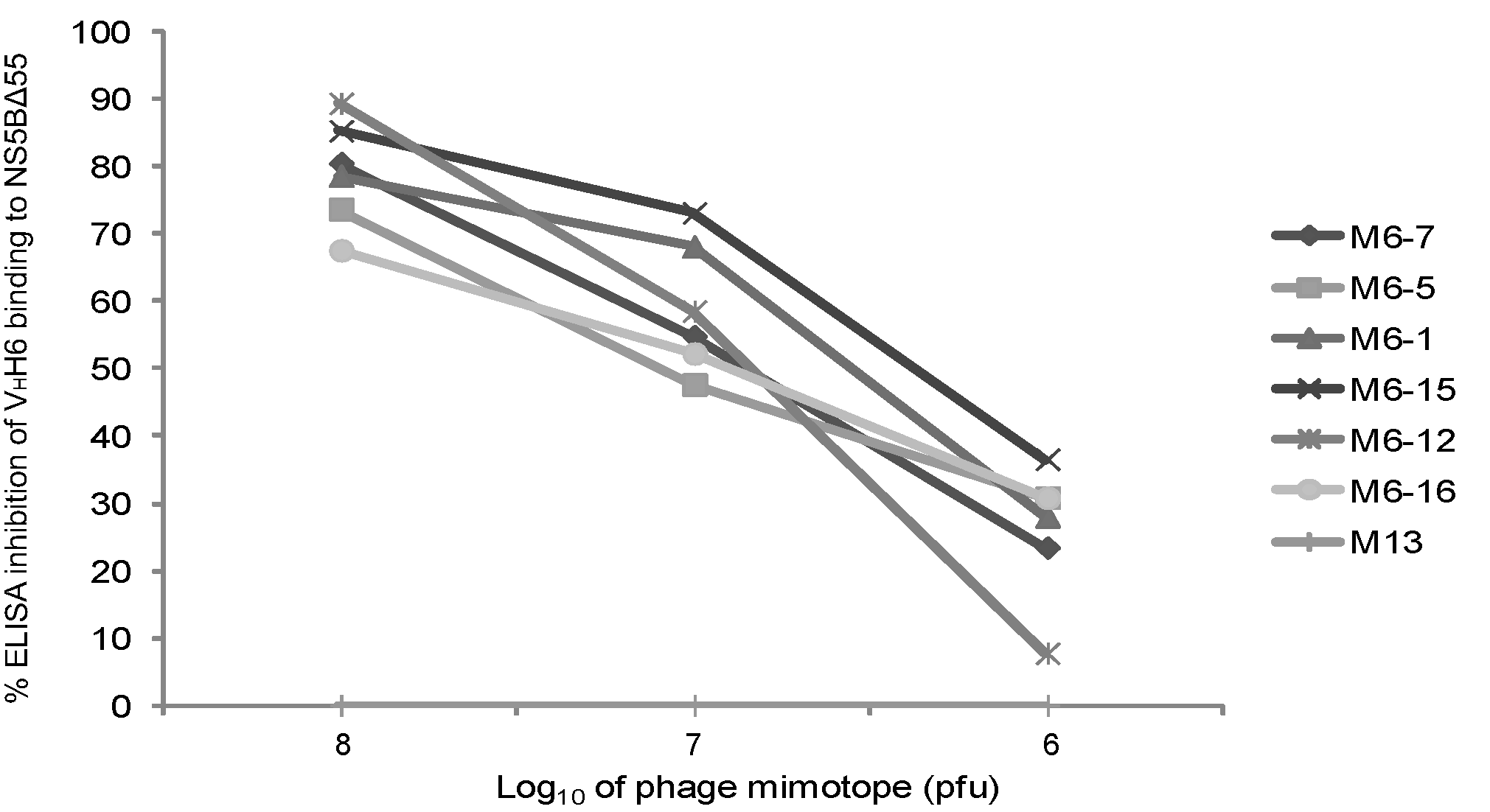

Supplement: Figure S2 — Percent ELISA inhibition of the VHH6 binding to the NS5BΔ55 mediated by the VHH6-phage mimotope groups 1, 2, 3 and 4. In the assay, phages displaying VHH6 mimotope group 1 (M6-7: ALWPPNLHAWVP), group 2 (M6-5: -FWSPN-HLMMNNL), group 3 (M6-1:–TLHLSHWTSSAL; M6-15: HYPTTQLPHHKQ) and group 4 (M6-12: GTVGRTEVSISE-; M6-16: -YSAHNYIGDSGR) (Table S1) at 106, 107 and 108 pfu were separately mixed with VHH6 (5 µg) before adding to the ELSA well containing immobilized NS5BΔ55 (10 µg). The % ELISA inhibition was calculated. The results indicate that the mimotopes could inhibit the VHH6 binding to NS5BΔ55 implying that the mimotopes carried the amino acid residues analogous to the native HCV NS5B polymerase which validated the mimotope search results. (TIF) [file pone.0049254.s002.tif]

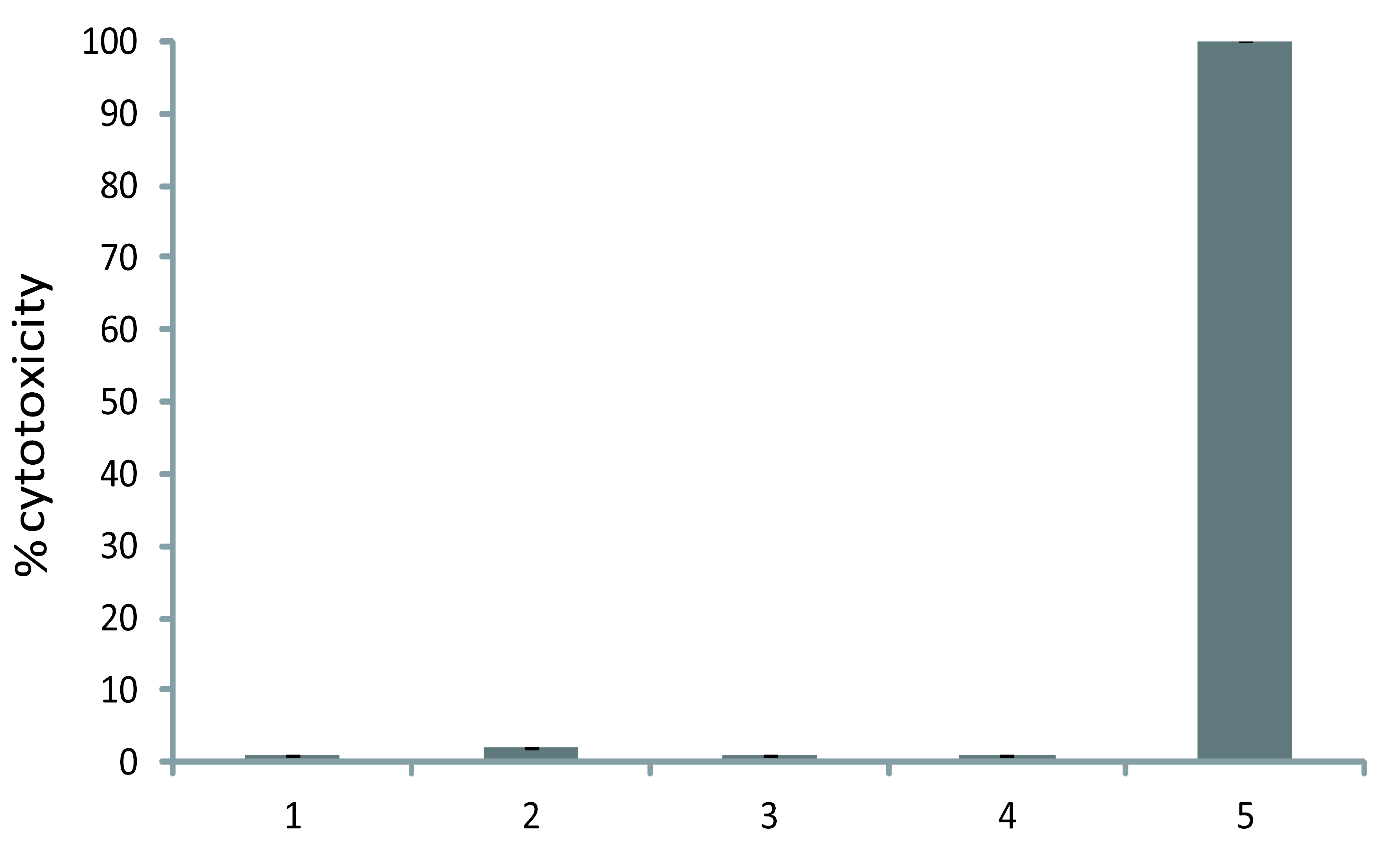

Supplement: Figure S3 — Results of LDH assay for determining cellular toxicity of 10 µM of VH9, VH13, VHH6 and VHH24 (1–4, respectively) on Huh7 cells after 24 hour incubation. Maximum LDH release control is shown in (5). All antibody preparations did not cause significant release of the LDH from the cells indicating the innocuousness of the preparations. (TIF) [file pone.0049254.s003.tif]

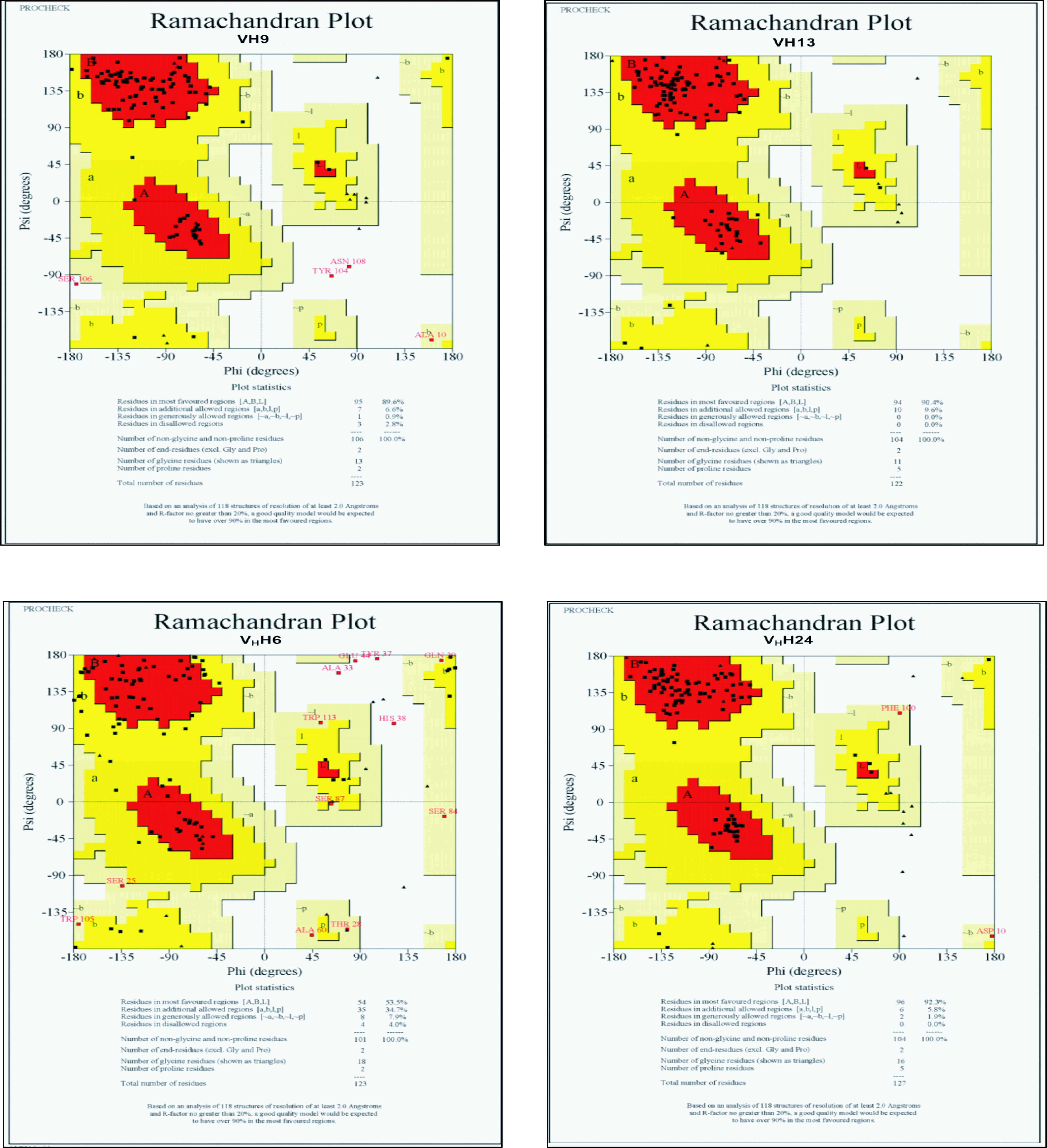

Supplement: Figure S4 — Ramachandran plots of the VH/VHH models. VH9, VH13, VHH6 and VHH24 had 2.8%, 0%, 4% and 0% of residue in disallowed region, respectively. The highest identity sequences used for protein homology modeling of VH9, VH13, VHH6 and VHH24 are sequences of PDB codes 2GCY, 2H32, 1VHP and 1F2X, respectively. (TIF) [file pone.0049254.s004.tif]
