# Supplementary material for: Cell Penetrable Humanized-VH/VHH That Inhibit RNA Dependent RNA Polymerase (NS5B) of HCV
Source: PLoS One. 2012 Nov 8;7(11):e49254. doi: 10.1371/journal.pone.0049254 (PMC3493538; doi:10.1371/journal.pone.0049254)
Supplement: Table S1 — Phage mimotope groups, respective 12-mer peptides displayed on the phages that bound to VH9, VH13, VHH6 and VHH24 and mimotope-matched peptides (tentative epitopes of the antibodies) on NS5B primary protein sequence. (DOC) [file pone.0049254.s005.doc]

Table S1. Phage mimotope groups, respective 12-mer peptides displayed on the phages that bound to VH9, VH13, V_H_H6 and V_H_H24 and mimotope-matched peptides (tentative epitopes of the antibodies) on NS5B primary protein sequence.

| **VH/V_H_H clone no.** | **Mimotope group** | **Mimotope sequence alignment** | **NS5B HCV Kalign (Accession no. YP_001491557.1)** |
| --- | --- | --- | --- |
| VH9 | 1 | M9-2 ALWPPNLHAWVP  M9-6 ALWPPNLHAWVP  M9-7 ALWPPNLHAWVP  M9-9 ALWPPNLHAWVP  M9-10 ALWPPNLHAWVP  M9-11 ALWPPNLHAWVP  M9-16 ALWPPNLHAWVP  M9-20 ALWPPNLHAWVP  ************ | 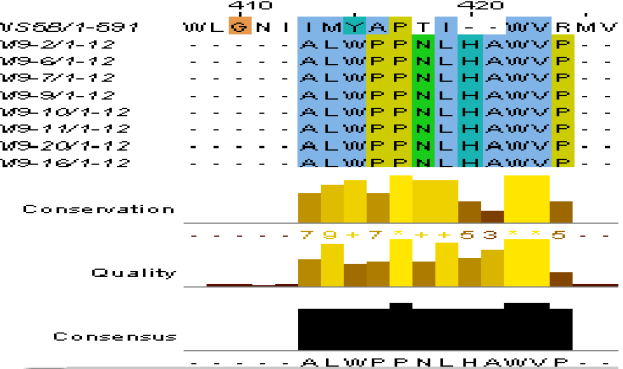 |
|  | 2 | M9-13 -WDLTSLFSRVSW  M9-15 HWWTSQPLS-YDY  * :. :* .: | 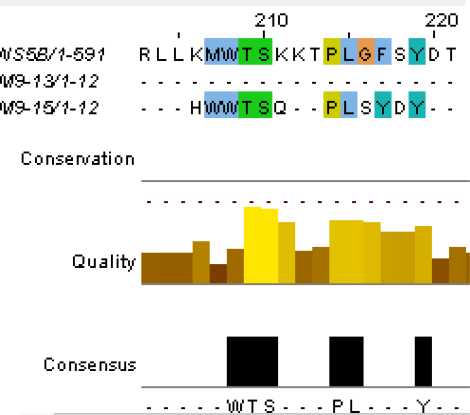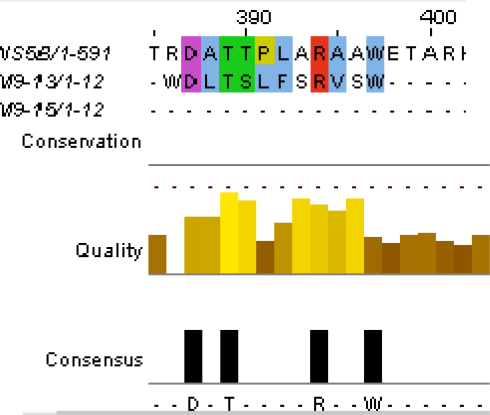 |

|  | 3 | M9-1 NHYLSYPSNPGI  M9-17 NHYLSYPSNPGI  ************ | 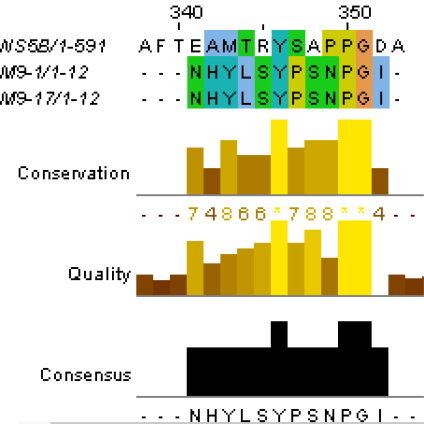 |
| --- | --- | --- | --- |
|  | 4 | M9-5 SVPSLKTWEQQQ  M9-12 LLSNHTTPYSRL  :.. .* .: | 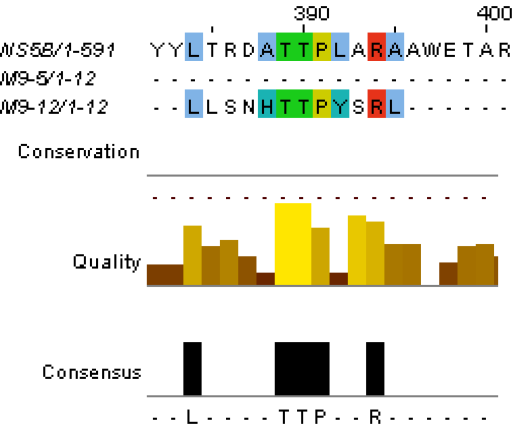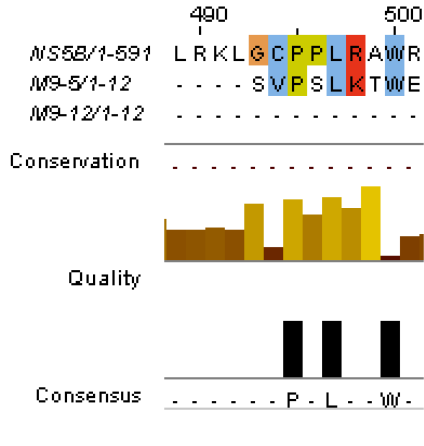 |

| **VH/V_H_H clone no.** | **Mimotope group** | **Mimotope sequence alignment** | **NS5B HCV Kalign (Accession no. YP_001491557.1)** |
| --- | --- | --- | --- |
| VH13 | 1 | M13-5 ALWPPNLHAWVP  M13-11 ALWPPNLHAWVP  M13-16 ALWPPNLHAWVP  ************ | 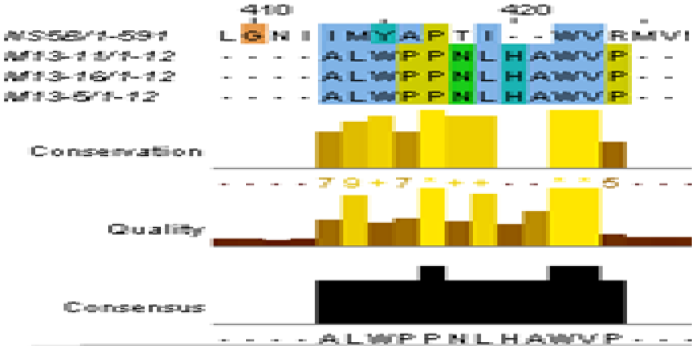 |
|  | 2 | M13-8 HHYNWNLPWLMS  M13-12 HYPTTQLPHHKQ  *: . :** . | 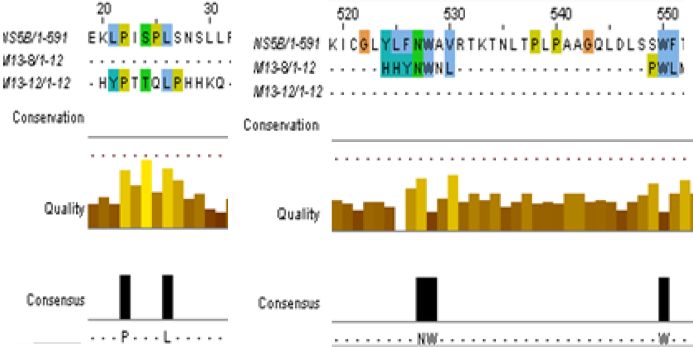 |
|  | 3 | M13-14 --GNHQSAWLQHRA  M13-9 VIGTPDSS—THGL  *. :*: * | 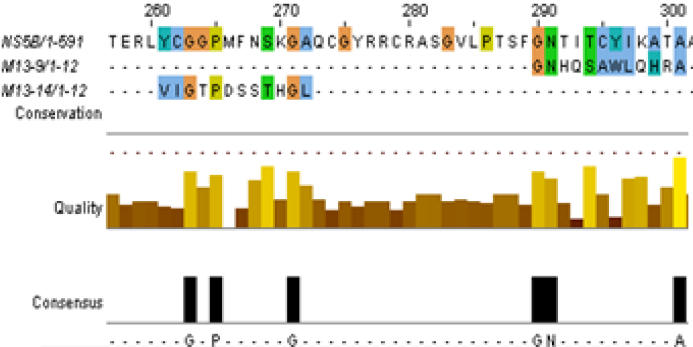 |

| **VH/V_H_H clone no.** | **Mimotope group** | **Mimotope sequence alignment** | **NS5B HCV Kalign (Accession no. YP_001491557.1)** |
| --- | --- | --- | --- |
| V_H_H6 | 1 | M6-7 ALWPPNLHAWVP  M6-9 ALWPPNLHAWVP  M6-10 ALWPPNLHAWVP  M6-13 ALWPPNLHAWVP  M6-17 ALWPPNLHAWVP  M6-18 ALWPPNLHAWVP  M6-19 ALWPPNLHAWVP  ************ | 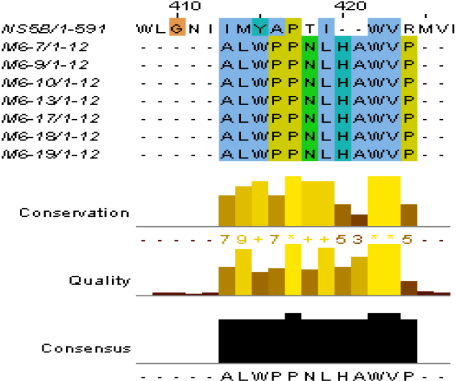 |
|  | 2 | M6-7 ALWPPNLHAWVP—  M6-9 ALWPPNLHAWVP—  M6-10 ALWPPNLHAWVP—  M6-13 ALWPPNLHAWVP--  M6-17 ALWPPNLHAWVP—  M6-18 ALWPPNLHAWVP—  M6-19 ALWPPNLHAWVP—  M6-5 -FWSPN-HLMMNNL  :*.** * : | 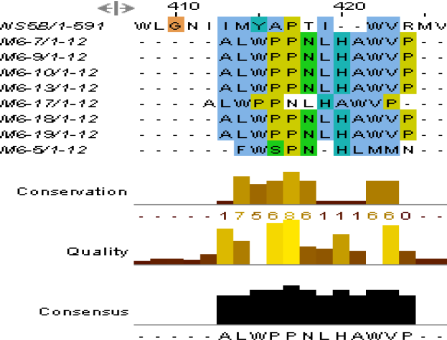 |
|  | 3 | M6-1 ---TLHLSHWTSSAL  M6-15 HYPTTQLPHHKQ---  * :*.* .. | 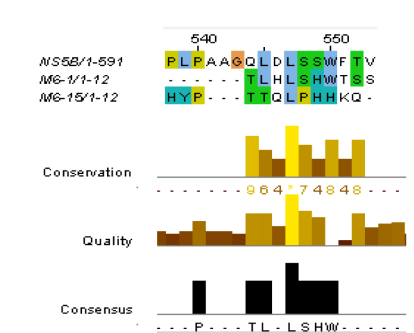 |
|  | 4 | M6-12 GTVGRTEVSISE-  M6-16 -YSAHNYIGDSGR  .:. :. * | 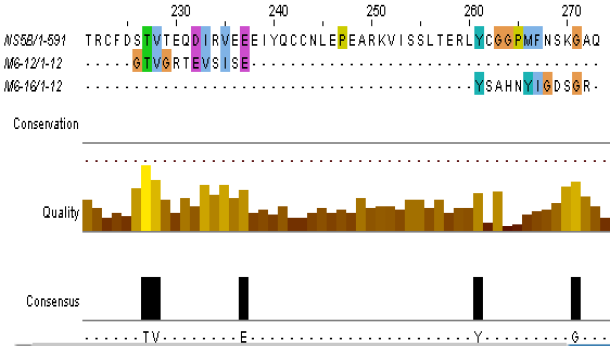 |

| **VH/V_H_H clone no.** | **Mimotope group** | **Mimotope sequence alignment** | **NS5B HCV Kalign (Accession no. YP_001491557.1)** |
| --- | --- | --- | --- |
| V_H_H24 | 1 | M24-1 YSAHNYIGDSGR  M24-5 YSAHNYIGDSGR  ************ | 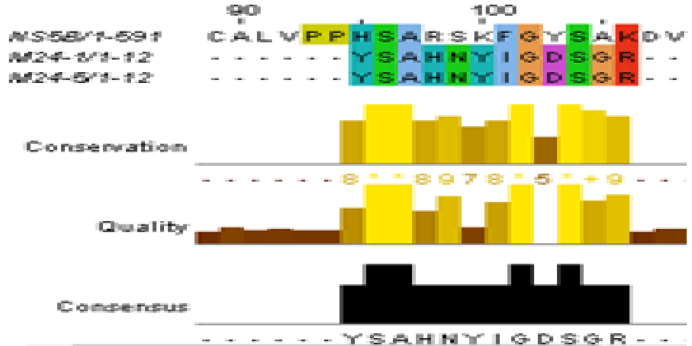 |
|  | 2 | M24-1 -YSAHNYIGDSGR  M24-5 -YSAHNYIGDSGR  M24-4 AHSANNFDVKGI-  :**:*: .. | 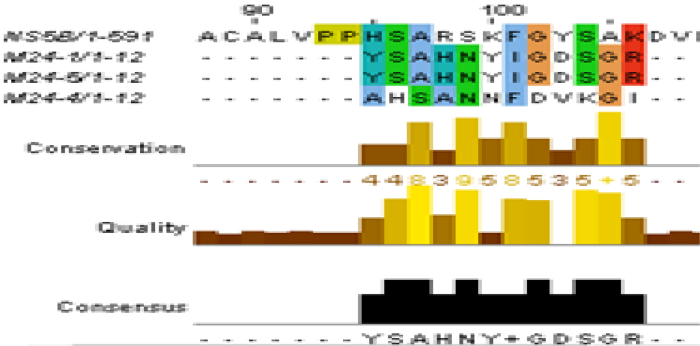 |
|  | 3 | M24-8 HWWHATTWQTQT  M24-15 FNSHDHTKQFGA  . * * * : | 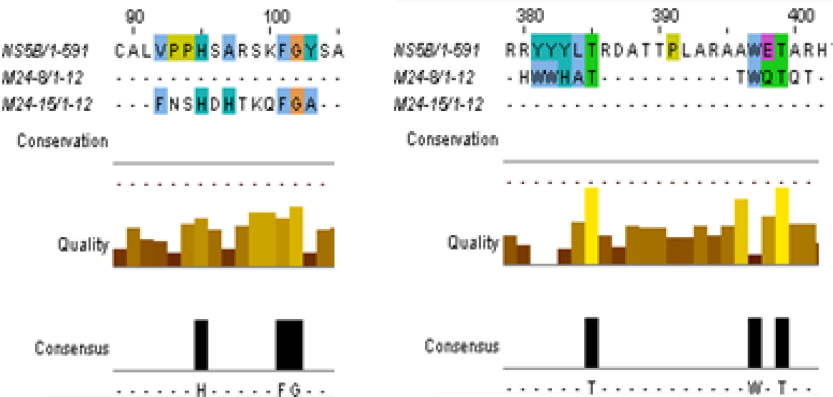 |
|  | 4 | M24-11 ALWPPILQAWAP  M24-13 ALWPPNLHAWVP  ***** *:**.* | 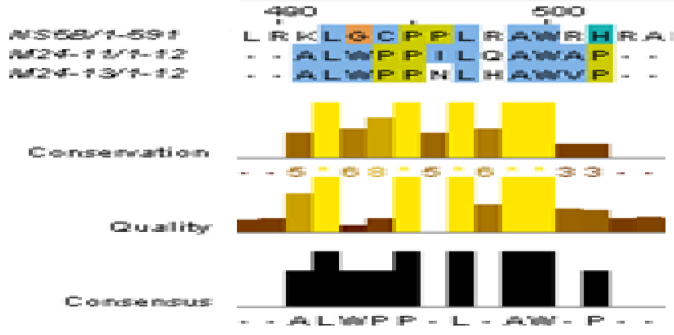 |
